# Giant Enhancement of Second-Harmonic Generation in Hybrid Metasurface Coupled MoS_2_ with Fano-Resonance Effect

**DOI:** 10.1186/s11671-022-03736-x

**Published:** 2022-10-04

**Authors:** Yunfei Xie, Liuli Yang, Juan Du, Ziwei Li

**Affiliations:** 1 College of Materials Science and Engineering, Hunan University, Changsha, 410082 Hunan People’s Republic of China; 2 State Key Laboratory of High Field Laser Physics, Shanghai Institute of Optics and Fine Mechanics, Chinese Academy of Science, Shanghai, 201800 People’s Republic of China; 3 School of Physics and Electronics, Shandong Normal University, Jinan, 250014 People’s Republic of China

**Keywords:** Metasurface, Second-harmonic generation, Fano-resonance, Nonlinear, Surface plasmon

## Abstract

Plasmonic nanostructures have been regarded as potential candidates for boosting the nonlinear up-conversion rate at the nanoscale level due to their strong near-field enhancement and inherent high design freedom. Here, we design a hybrid metasurface to realize the moderate interaction of Fano resonance and create the dual-resonant mode-matching condition to facilitate the nonlinear process of second harmonic generation (SHG). The hybrid metasurface presents dipolar and octupolar plasmonic modes near the fundamental and doubled-frequency wavelengths, respectively, further utilized to enhance the SHG of low-dimensional MoS_2_ semiconductors. The maximum intensity of SHG in hybrid metasurface coupled MoS_2_ is more than ten thousand times larger than that of other structure-units coupled MoS_2_. The conversion efficiency is reported to be as high as 3.27 × 10^−7^. This work paves the way to optimize nonlinear light–matter interactions in low-dimensional structures coupled with semiconductors.

## Introduction

Second-harmonic generation (SHG) is a kind of nonlinear optical process. Two photons of the same frequency coupled in nanostructures result in a single-photon emission with twice the fundamental frequency. System ground state information and pump light polarization are recorded via SHG signals, which have been used extensively in lasers [1, 2], holographic images [3], and measurements of ultrashort-width pulses [4, 5], as well as medical imaging applications [6]. In most nonlinear materials, the nonlinear optical conversion is limited due to weak optical responses and light–matter interactions. Although it can be improved in materials with a "long" light–matter interaction length satisfying phase-matching conditions or under a strong localized field, it is usually unavailable in low-dimensional materials or hybrid nanostructures at the nanoscale.

A possible strategy to tackle this problem is to design hybrid nanostructures interacting with nonlinear materials [7, 8]. Electromagnetic (EM) fields can be localized in the near-field and confined to a very tiny mode volume by photonic nanostructures resonating with distant excitations (which means longer interaction length of light at particular dimensions) [9]. This kind of resonant nanostructures permits the flexible design without considering the phase-matching criteria, but the rational design of structures producing specific electromagnetic mode matching is required. It has been claimed that the SHG process can be significantly boosted by matching their localized electromagnetic resonance with either the excitation or the emission wavelength in both dielectric and plasmonic nanostructures [10, 11].

Plasmonic Fano resonance interferes with bright superradiant and dark subradiant modes, providing a platform for ultrasensitive signal detection applied in chemistry, physics, and biological sciences [12, 13]. Generally, characteristic peaks or dips in spectra indicate specific EM resonance modes. A narrow dip in the scattering spectrum of a plasmonic Fano resonant system results in the suppression of radiative losses, making it valuable in promoting the nonlinear conversion efficiency of nanoscale semiconductors. For this reason, the studies on the nonlinear responses and improved light–matter interactions in Fano resonant nanostructures have been the subject of extensive research [14, 15]. On the bases of plasmonic metamolecule, magnetic Fano resonance-induced SHG enhancements have been reported by modulating the excitation wavelength at the magnetic Fano dip of the plasmonic structures [16, 17]. Gold nanostructures with doubly resonant mode matching at the excitation and second harmonic (SH) wavelengths have been reported to achieve a nonlinear coefficient of ∼ 5 × 10^–10^ W^–1^ for SHG [18]. Compared with the GaAs nanodisk metasurface, a Fano resonance metasurface composed of broken symmetric resonators can achieve multiple enhancements [19].

Here, we propose a hybrid metasurface (a paired bull-eye rings locating at the center of a wall ring) having mode matching and plasmonic Fano resonance to enhance the SHG of low-dimensional MoS_2_ semiconductors. The hybrid metasurface is designed to behave with dual-resonance modes overlapping the fundamental and the SH wavelengths. Simulations of charge distribution at the doubled-frequency reveal that the hybridized Fano resonance mode has been established. The intensity of the SHG signal of hybrid metasurface coupled MoS_2_ is more than ten thousand times larger than that of metasurface units coupled MoS_2_ due to the combination of mode-matching conditions and moderate-interaction Fano resonance. Furthermore, the conversion efficiency of the proposed configuration is detected to be as high as 3.27 × 10^–7^. The hybrid metasurface proposed in this work may pave the way to explore the enhancement of nonlinear optical processes by inducing Fano resonance and mode matching conditions.

## Results and Discussion

**Fig. 1:**
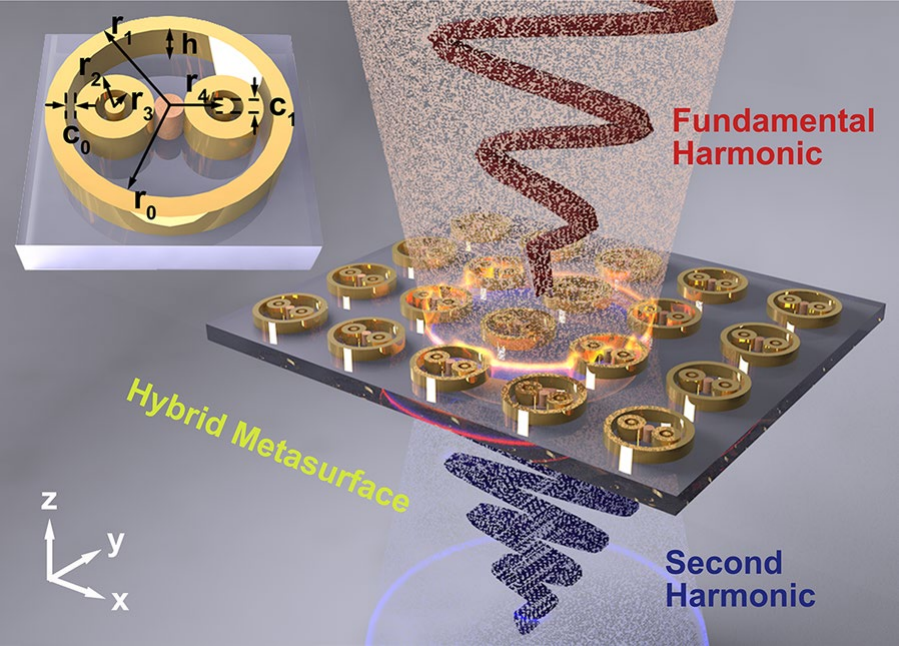
Schematic view of SHG induced by hybrid metasurface with a wall ring and paired bull-eye rings. The left corner inset shows a unit cell of the metasurface with geometrical parameters of ${r}_{1}$ = 124 nm, ${r}_{0}$ = 105 nm, ${r}_{2}$ = 40 nm, ${r}_{3}$ = 20 nm, ${r}_{4}$ = 55 nm, $h$ = 80 nm, ${c}_{0}$ = 10 nm, ${c}_{1}$ = 20 nm

Figure 1 shows the schematic view of a hybrid metasurface with a MoS_2_ semiconductor located at the structure center. The generation and enhancement of SHG are realized by designing the plasmonic resonant modes matching the excitation wavelengths. Paired bull-eye rings are employed inside a wall ring to achieve stronger Fano resonance. Where eight parameters, namely, can characterize the structure geometry, the inner and outer radii of the wall ring are ${r}_{0}$ and ${r}_{1}$, respectively. Similarly, the inner and wall radii of bull-eye rings are ${r}_{2}$ and ${r}_{3}$, respectively. The radius and the distance from the center of the bull-eye disk are ${r}_{4}$ and ${c}_{1}$, respectively. The gap of the bull-eye and wall ring is denoted by ${c}_{0}$, while $h$ is the thickness of the gold layer. MoS_2_ semiconductor is in the gap between the paired bull-eye rings, and its size is chosen to ensure that they contact the bull-eye rings. The hybrid metasurface can be experimentally fabricated using the “Sketch and Peel” lithography to realize the high-resolution patterning [20].

The Total-field scattered field (TFSF) as an incident light source is set to be linearly polarized parallel to the *x*–*z* plane and the coaxial direction of bull-eye rings [21]. In our work, simulation is carried out by using a commercial Lumerical software package [22]. Perfectly matched layers (PML) were set in all simulation directions. The complex dielectric constant of gold [23] and the refractive index of MoS_2_ [24] was taken from public experimental data and fitted. Similarly, SiO_2_ with refractive index of 1.5 is set be the substrate and support of the device.

**Fig. 2:**
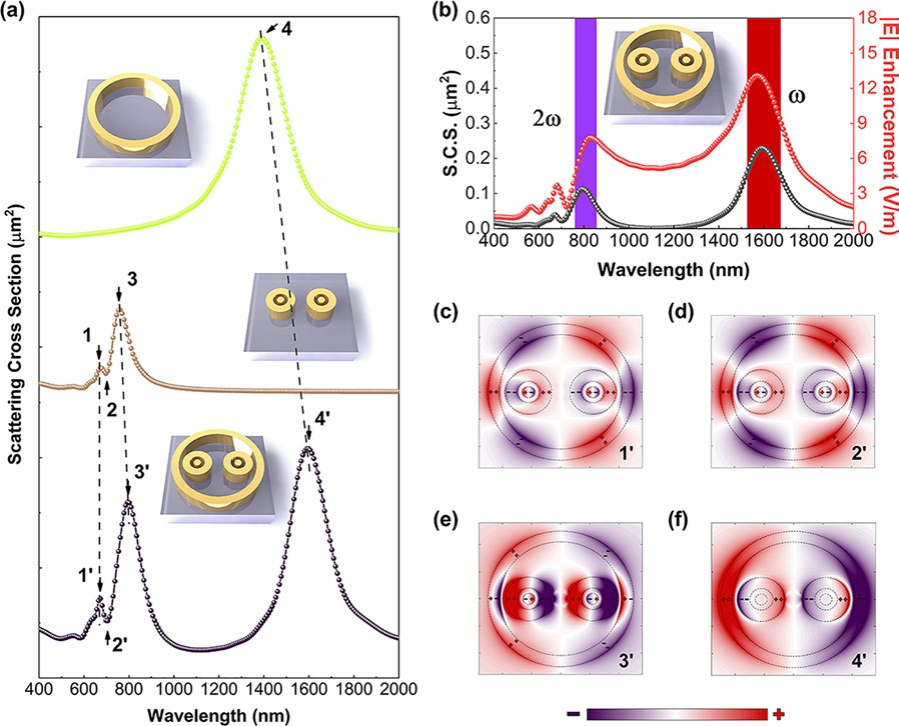
SCSS of designed structures and their characteristic charge distributions. **a** SCSS of a wall ring, paired bull-eye rings, and the hybrid metasurface. Insets are the schematics of simulated structures. **b** SCSS (gray line) of the hybrid metasurface and the near-field enhancement (red line) at the center. The spectrum shows a simultaneous pronounced over-lap with the fundamental and SHG wavelengths, which are marked by red and purple stripes. The inset shows a unit of the hybrid metasurface. **c**–**f** Charge distributions at featured peaks of 1′–4′

To satisfy the mode-matching conditions, the resonance of the hybrid metasurface needs to be specifically optimized for both fundamental and SHG wavelengths. Scattering cross sections spectrum (SCSS) was simulated to discuss the plasmonic modes induced by structure units. Figure 2a compares the SCSS of three nanostructure units, where the strong scattering peak (peak 4) is shown at the fundamental wavelength (1450 nm), and featured Fano resonance peaks (peaks 1–3) have appeared in the SCSS of paired bull-eye rings at 650–800 nm of the wavelength spectrum. Fano resonance is formed by the moderate interaction of bright superradiant and dark sub-radiant, corresponding to characteristic peaks (peaks 1 and 3) and a narrow dip (peak 2). When a wall ring is further induced in paired bull-eye rings, the SCSS exhibits double resonances, including fundamental mode matching at 1600 nm and Fano resonance at 650–850 nm. Dipolar resonance contributes to the peak at the fundamental wavelength while the resonance is simultaneously tuned to match the SHG emission wavelength. All peaks in SCSS are shifted compared to paired bull-eye rings, which is influenced by the gap resonance between paired bull-eye structures and the wall ring. The corresponding shifted peak is noted as peaks 1′–4′.

The bi-coordinate of the *y*-axis layout of Fig. 2b depicts the relationship between SCSS and near-field enhancement intensity $\left|E\right|$ at the structure center. The peak position of the Fano resonance is highly related to the field enhancement of the subradiation mode [25]. The resonance mainly happens at both fundamental and Fano resonance wavelengths. Figure 2c–f shows mapping images of charge density distribution corresponding to the spectral features of 1′–4′ in Fig. 2a. In Fig. 2c, six nodes and two nodes on the wall ring and bull-eye rings, respectively, exhibit a hybridized octupolar mode. The featured dip peak 2′ is quite close to peak 1′, and their charge distributions are quite similar, as shown in Fig. 2d. For peak 3′, the enhanced charge distribution intensity elucidates a stronger peak in SCSS. The region of positive and negative charge reversed due to Fano resonance. Intriguingly, both the charge distributions on the wall ring and bull-eye ring at peak 4′ exhibit two nodes, as depicted in Fig. 2f, from which the superradiant mode results.

**Fig. 3:**
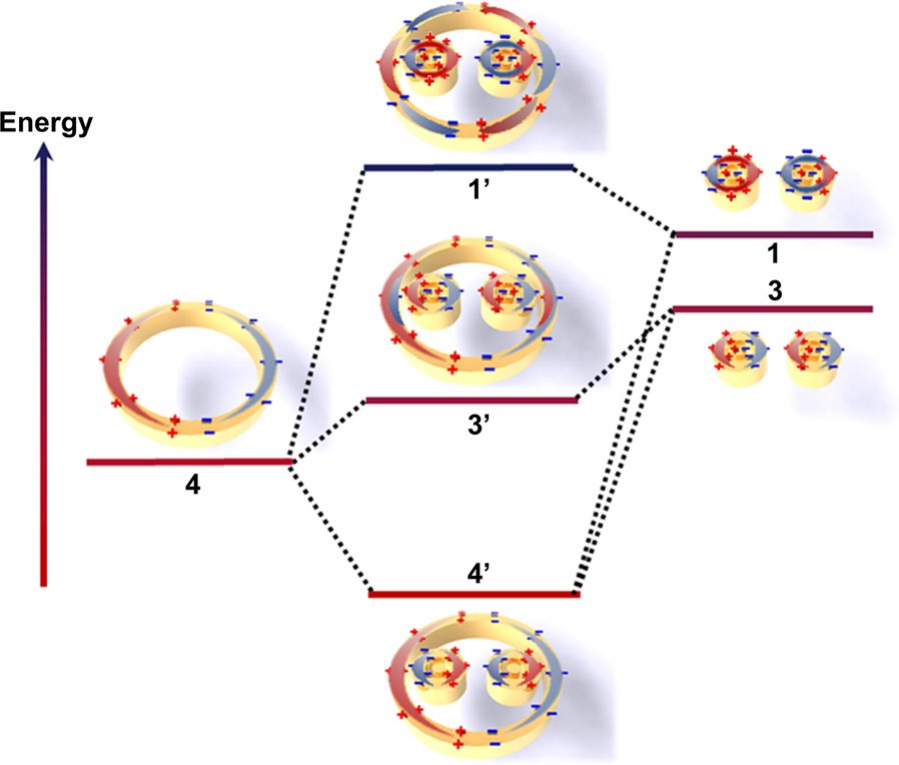
Schematic of plasmonic hybridization between the dipolar mode of the wall ring structure and the hexapole mode of paired bull-eye rings, and the energy diagram of the degenerated plasmon bright and dark modes

To comprehend the origin of this Fano resonance, the primary plasmon modes of metasurface units should be first understood. In Fig. 3, a plasmon hybridization model plotted in an energy diagram is established to understand the mode changes. Wall-ring unit (peak 4) and the bull-eye rings (peak 3) are directly excitable resonant modes of the bonded dipole plasmon, and both have bright modes and superradiant states. In contrast, another mode of the bull-eye rings (peak 1) is a low-emission dark quadrupole mode due to dipole excitation of plasmon interaction between the two cavities. The coupling between the dark state plasmons of the bull-eye rings results in the development of dark mode and subradiative state of the bound quadruple ring mode. In the unit of bull-eye rings, the outer ring and the inner disk form a bonding (peak 1) and an anti-bonding mode (peak 3) after mode hybridization [21, 26]. The bonded quadrupole ring mode occurs simultaneously with the bright-state superradiant state, and the destructive interference between them induces the formation of the Fano resonance in the bull-eye rings. After a further complex coupling of two units, hybrid plasmon modes 1′–4′ are constructed. Due to the hybridization effect, the energy level of mode 4′ becomes lower, while for modes 1′ and 3′ it shifts toward higher and lower energies distinctly compared to their original modes. The complex modes of 1′ and 3′ are bonding and antibonding hybridized octupole modes. The bonded octupole mode occurs simultaneously with the bright-state superradiant state, and the destructive interference between them leads to the formation of Fano resonances in the metasurface.

To discuss the nonlinear process of SHG from a hybrid metasurface coupled MoS_2_ semiconductor, the SHG enhancement factor (*τ*) is introduced to describe the enhancement intensity, which is defined as the ratio of the SHG intensity converted from the metasurface to the intensity of the MoS_2_ nanoparticles. The volumetric effective second-order nonlinear polarizability 0.78 pm V^−1^ is used to simulate SHG emission from gold [27], and the similarly, the second-order magnetic susceptibility of MoS_2_ chose to be 0.25 nm V^−1^ [28]; both consist with previously reported values [29]. In simulations, the hybrid metasurface is irradiated with a normal incident Gaussian pulse with a field amplitude of 1.55 × 10^8^ V m^−1^, and the corresponding peak field strength is 3.2 GW cm^−2^, which is less than the damage threshold of gold [30]. A narrow-band Gaussian pulse with a full width at half a maximum of 150 fs and an initial time delay of 400 fs is set to excite the designed hybrid metasurface, corresponding to a spectral width of 22.05 nm after Fourier transform.

**Fig. 4:**
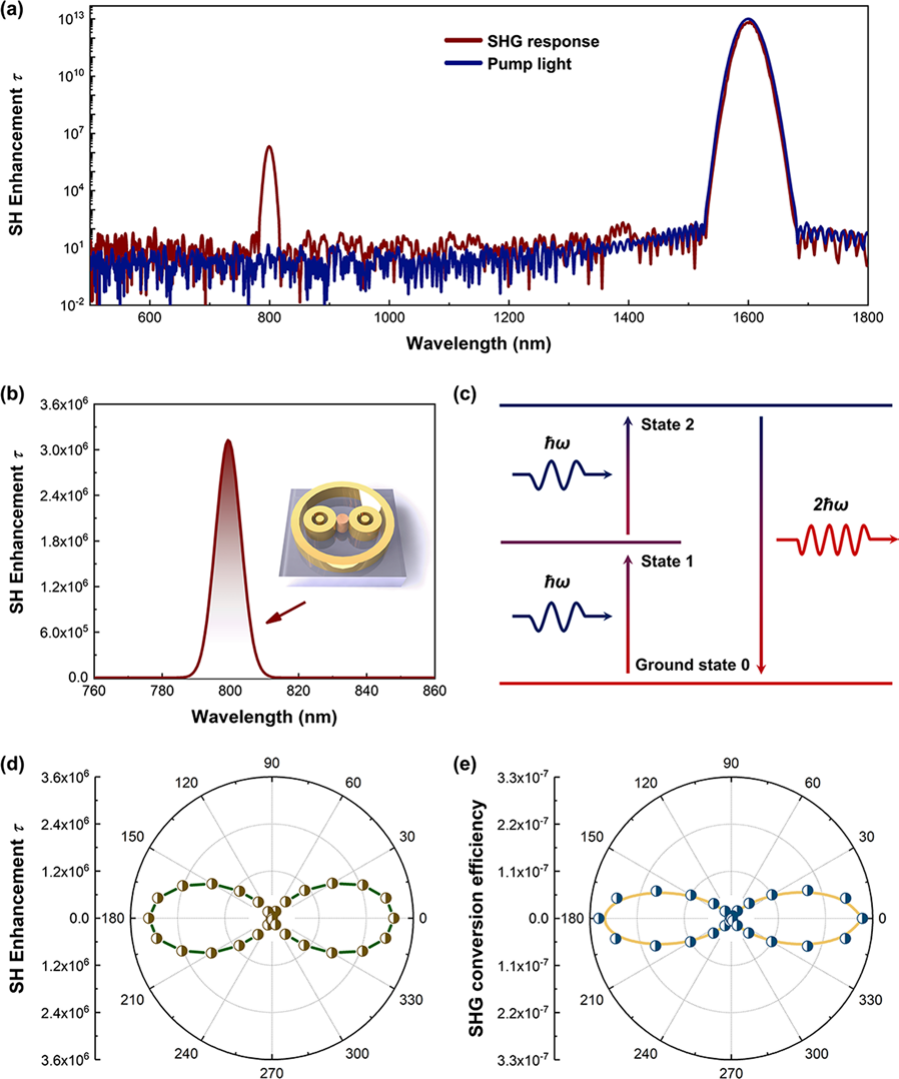
Study of SH enhancement. **a** Log-plot spectra of SHG signal (red line) as defined by the SHG enhancement factor *τ*. The blue-dashed line represents the pump light with the same processing. **b** Characteristic SHG peak of metasurface coupled MoS_2_ centered at 800 nm. The inset shows a unit of the hybrid metasurface and MoS_2_. **c** Scheme of the fundamental dipole transitions involved in the multi-resonant plasmon-induced SHG process. **d** Polar diagram of the polarization of the generated SHG signal from the hybrid metasurface. **e** Polar diagram of the polarization of the generated SHG signal conversion efficiency from the hybrid metasurface

Figure 4a shows the SHG enhancement factor (*τ*) of the hybrid metasurface (red line), while the blue line corresponds to the ratio of pump light to SHG from MoS_2_ nanoparticles. The basic femtosecond excitation at 1600 nm produces an instantaneous SHG response centered at 800 nm. As shown in Fig. 4b, SHG response is amplified near the wavelength of 800 nm. The SHG conversion efficiency is defined as ${\eta }_{\mathrm{SH}}$ = ${P}_{\mathrm{SH}}$/${P}_{\mathrm{FH}}$, where ${P}_{\mathrm{FH}}$ = $I_{{{\text{FH}}}} \cdot A$ represents the power at the fundamental wavelength, ${I}_{\mathrm{FH}}$ is the pump intensity, $A$ is the conversion efficiency per unit area, and ${P}_{\mathrm{SH}}$ is the power at the SHG wavelength [7]. The calculated SHG conversion efficiency of the hybrid metasurface is up to 3.27 × 10^−7^. Figure 4c shows the energy diagram of SHG, a coherent three-wave mixing process, where the absorption of two photons (*ħω*) at the excitation wavelength assisted by a first plasmonic mode, results in a radiative frequency-doubled photon (2*ħω*) assisted by a second plasmonic mode, restoring the system ground state through the emission of a SH photon [18]. The SHG efficiency depends on the resonant EM-field intensity both at the fundamental and the doubled-frequency wavelength. Thus, it requires the optimization of structural geometry to meet the demand of frequency matching and spatial overlap-integral between modes [31, 32]. Fano resonance can generate moderate coupling to enhance the local field enhancement in nanoscale volume, which is an alternative and essential to optimizing SHG conversion efficiency.

In order to study the influence of incident light polarization on SHG intensity, the polarization distribution of the radiated SHG signals is plotted in Fig. 4d. As shown, when the incident light is parallel to the *x* direction (the polarization angle *θ* = 0°), the largest SHG signal is obtained while the intensity of the SHG signal gradually decreases as the direction of the incident light moves away from *x* to the *y*-direction. Furthermore, when the polarization direction of the incident light is parallel to the *y* direction (the polarization angle *θ* = 90°), the SHG signal almost becomes zero. Thus, it clearly reveals that the SHG signal from the hybrid metasurface strongly depends on the polarization of the incident light. The polarization diagram of the SHG signal conversion efficiency generated by the hybrid metasurface is shown in Fig. 4e. The SHG signal conversion efficiency shows a similar polarization response to incident light; for instance, the SHG signal conversion efficiency at *θ* = 0° reaches the maximum value, while for *θ* = 90° the conversion efficiency declines to a minimum value.

**Fig. 5:**
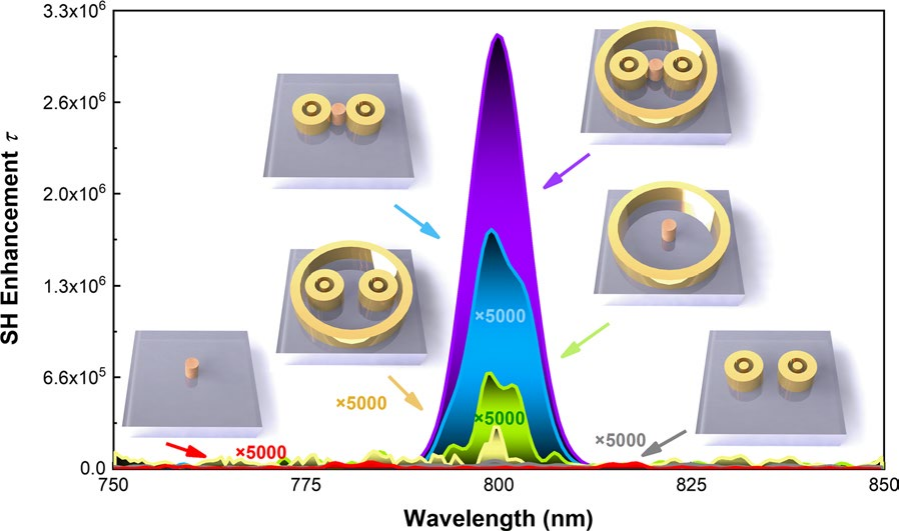
SHG enhancement of four different structures. Insets show corresponding schematics, including pure MoS_2_ nanoparticles, paired bull-eye rings without MoS_2_, hybrid metasurface without MoS_2_, wall ring structure, paired bull-eye rings, and hybrid metasurface

At the end of this discussion, here is a comparison (Fig. 5) of SHG enhancement of six different structures. At first, there is almost no SHG signal was found for pure MoS_2_ nanoparticles (red line), but the maximum intensity of SH enhancement appears to be 1.31 × 10^2^ when the MoS_2_ nanoparticle is located at the center of the wall ring. The SH enhancements of paired bull-eye rings and designed hybrid structure without MoS_2_ are nearly zero and 60, respectively. In comparison, the maximum intensity of SH enhancement of bull-eye coupled MoS_2_ nanoparticle gets 2.7 times larger than that of wall ring coupled MoS_2_ nanoparticle. Remarkably, in hybrid metasurface-coupled MoS_2_ nanoparticles, the intensity is increased to 3.13 × 10^6^, which is more than 10^4^ times larger than other structure units coupled MoS_2_ nanoparticles. A previous report shows that the conversation efficiency of MoS_2_ monolayer enhanced by Au@SiO_*x*_ nanoparticles is 1.24 × 10^−7^ at the 100 GW cm^−2^, and the SHG enhancement is 1.88 detected from experiments [33]. Another works demonstrate that SHG enhancements of monolayer MoS_2_ on suspended metallic nanostructures are 1528 in experiments and 1898 in simulations, respectively [34]. Our designed hybrid nanostructure has great advantage and potential in realizing SHG enhancement of low-dimensional materials.

Although hybrid plasmonic nanostructures with Fano resonant modes have been widely reported, the enhancement efficiency of SHG is still limited by finite electromagnetic field enhancement. In a fixed geometric dimension, the rational design of abundant plasmonic gaps can induce more “hot spots” creating stronger total-field-enhancement for SHG enhancement. Besides, the high-order plasmonic modes in our designed nanostructures can suppress the radiation loss and provide sharper line shape than dipole modes, which may help to increase the coupling sensitivity at resonant wavelengths. This rational design of plasmonic metasurface inducing matching conditions and Fano resonance can effectively enhance the SHG signals and conversion efficiency.

## Conclusion

In summary, this contribution provides an understanding of a hybrid metasurface to realize the moderate interaction of Fano resonance and create the dual-resonant mode-matching condition to facilitate the nonlinear process of SH generation. Our hybrid metasurface presents the dipole and octupole plasmonic modes at the fundamental and doubled-frequency wavelengths. Simulations of charge distribution at the doubled-frequency reveal the hybridized Fano resonance mode formation. The designed metasurface produces a conversion efficiency of 3.27 × 10^–7^. In this work, the SHG signal of hybrid metasurface coupled-MoS_2_ nanoparticle is more than ten thousand times larger than that of other metasurface unit coupled MoS_2_ nanoparticles. The combination of mode-matching conditions and moderate interaction of Fano resonance can enhance SHG conversion efficiency effectively. The related concept may also be applicable to other nonlinear processes, such as third harmonic generation, sum-frequency generation (SFG), and difference frequency. This paves the way to achieve infrared frequency up-conversion and overcome difficulties in infrared reception. Furthermore, combined with frequency doubling and frequency mixing technologies, it can achieve modulation in a wide range from infrared to ultraviolet regimes. Our designed hybrid metasurface opens a new avenue for optimizing nonlinear light–matter interactions in low-dimensional semiconducting materials and nanostructures.

## Data Availability

All data generated or analyzed during this study are included in this published article.
